# *Bacillus cereus* ATCC 14579 RpoN (Sigma 54) Is a Pleiotropic Regulator of Growth, Carbohydrate Metabolism, Motility, Biofilm Formation and Toxin Production

**DOI:** 10.1371/journal.pone.0134872

**Published:** 2015-08-04

**Authors:** Hasmik Hayrapetyan, Marcel Tempelaars, Masja Nierop Groot, Tjakko Abee

**Affiliations:** 1 Laboratory of Food Microbiology, Bornse Weilanden 9, 6708 WG Wageningen University, Wageningen, The Netherlands; 2 Top Institute of Food and Nutrition (TIFN), Nieuwe Kanaal 9A, 6709 PA, Wageningen, The Netherlands; 3 Food and Biobased research, Wageningen UR, Bornse Weilanden 9, 6708 WG, Wageningen, The Netherlands; Loyola University Chicago, UNITED STATES

## Abstract

Sigma 54 is a transcriptional regulator predicted to play a role in physical interaction of bacteria with their environment, including virulence and biofilm formation. In order to study the role of Sigma 54 in *Bacillus cereus*, a comparative transcriptome and phenotypic study was performed using *B*. *cereus* ATCC 14579 WT, a markerless *rpoN* deletion mutant, and its complemented strain. The mutant was impaired in many different cellular functions including low temperature and anaerobic growth, carbohydrate metabolism, sporulation and toxin production. Additionally, the mutant showed lack of motility and biofilm formation at air-liquid interphase, and this correlated with absence of flagella, as flagella staining showed only WT and complemented strain to be highly flagellated. Comparative transcriptome analysis of cells harvested at selected time points during growth in aerated and static conditions in BHI revealed large differences in gene expression associated with loss of phenotypes, including significant down regulation of genes in the mutant encoding enzymes involved in degradation of branched chain amino acids, carbohydrate transport and metabolism, flagella synthesis and virulence factors. Our study provides evidence for a pleiotropic role of Sigma 54 in *B*. *cereus* supporting its adaptive response and survival in a range of conditions and environments.

## Introduction

Microorganisms may encounter many different environments during their lifecycle and rapid adaptation to these specific conditions requires tailored regulatory mechanisms. As part of such a mechanism, alternative sigma factors regulate the onset and expression level of a subset of genes at a given physiological state of the cell. There are different types of sigma factors in bacteria, each of them regulating a specific response, such as Sigma B regulating gene expression in response to a range of stresses including heat, low temperature and acid in *Bacillus subtilis* and related species [1, 2] and for example Sigma 32 that supports heat shock survival in *Escherichia coli* [3]. The majority of these sigma factors belong to the Sigma 70 family, with Sigma 54 as an exception, and as the only known representative of this class [4]. Sigma 54, encoded by the *rpoN* gene in *B*. *cereus*, also referred to as SigL in *B*. *subtilis* [5] and RpoN in *E*. *coli* [6], is unique among all sigma factors in its structure and the mechanism of action since it needs specific activator proteins called Enhancer Binding Proteins (EBPs) and ATP hydrolysis in order to initiate transcription [7]. Specificity and activity of Sigma 54 dependent transcription is modulated by EBPs which bind to specific enhancer binding sequences and are triggered by different environmental cues [8].

The Sigma 54-controlled functions described for bacterial species are diverse and include nitrogen metabolism [5, 9], motility [10, 11], biofilm formation [12], stress tolerance and resistance to antimicrobials [13, 14], carbohydrate uptake and metabolism [15], and virulence [16]. In *B*. *subtilis* Sigma 54 (SigL) is known to be involved in regulation of cold shock adaptation [17], degradation of the branched chain amino acids isoleucine and valine [18] and acetoin catabolic pathway [19], whereas in *Bacillus thuringiensis* regulation of the *gab* gene cluster, involved in γ-aminobutyric acid (GABA) shunt which can channel glutamate into the tricarboxylic acid (TCA) cycle has been reported [20, 21]. A recent study showed a role of Sigma 54 in nitrogen utilisation in *B*. *thuringiensis* and proposed putative regulon members for this sigma factor [22].

A cross-phylum *in silico* analysis by Franke et al. [23] aiming for prediction of Sigma 54 functions based on its genetic context, associated EBP-activators and promoters, and reported phenotypes, revealed a role for Sigma 54 as a modulator of bacterial cell exterior as a unifying theme. This control is executed by regulating the transport and biosynthesis of components involved in the interaction of the cell with its environment, such as the cell wall, flagella, extracellular polysaccharides and proteins. For pathogens such as for example *Bacillus cereus*, this may imply a role of this sigma factor in host colonization and biofilm formation [23, 24].

*Bacillus cereus* is a foodborne pathogen that is ubiquitously present in the environment showing high capacity to adapt to different environmental niches. Soil is the main reservoir of *B*. *cereus* spores and food can serve as a vehicle to transfer them to the host. Apart from food spoilage, this species may cause food poisoning due to production of toxins. The spores of *B*. *cereus* survive many stresses applied by food producing industries such as heat and disinfectant treatments, making them hard to eliminate as contaminants. Surviving *B*. *cereus* spores germinate and grow out into vegetative cells, which can also cope with unfavourable conditions, such as anaerobic environment [25, 26], acidic environments [27] or low temperature [28].

The aim of this study was to assess the role of Sigma 54 in *B*. *cereus* by a comparative transcriptomic and phenotype analysis, using *B*. *cereus* ATCC 14579 wild type, its marker-less *rpoN* deletion mutant, and a complemented strain under different growth conditions.

Presented data shows that the *B*. *cereus rpoN* mutant was impaired in many different cellular functions which were correlated with differences in gene expression. This provides evidence for a pleiotropic role of Sigma factor 54 in *B*. *cereus* supporting adaptive responses and performance of this foodborne pathogen in a range of conditions and environments.

## Materials and Methods

### Strains and culture conditions

*Bacillus cereus* ATCC 14579 wild type (WT), its *rpoN* mutant derivative (*ΔrpoN*) and complemented strain (*ΔrpoN*-comp) stocks from -80°C were streaked on BHI (Brain Heart Infusion, Becton Dickinson) plates and incubated for 24 h at 30°C. Single colonies were inoculated into BHI broth and cultivated for 18 h at 30°C with aeration (200rpm). Erythromycin (10 ug/ml) was added for the *rpoN*-comp strain to agar plates and overnight culture broth.

Occasionally, after prolonged static incubations (>48h) a spontaneous secondary mutant with a more widespread and round colony appeared. To avoid interference of this secondary mutant, incubation times were kept within 48 h and routinely, cultures were screened on plate to confirm presence of a single colony morphology. Interestingly, a similar phenomenon has been described previously for a *Pseudomonas fluorescence rpoN* mutant [29].

### Growth, sporulation and diarrhoeal enterotoxin production

Aerated (200 rpm) and static growth of the strains was monitored at 30°C in 250 ml erlenmeyer flasks filled with 50 ml BHI. Anaerobic cultivation was performed in rubber sealed serum bottles which were flushed with nitrogen for 2 h before inoculation. Nitrogen flushing was repeated for 20 sec. after each sampling point to ensure anaerobic conditions after sampling. Anaerobic cultures were incubated at 200 rpm. At regular time points between 0 and 48 h, samples were taken and the OD at 600 nm was measured.

The number of spores in the aerated culture was determined by plating the samples after heating for 10 min at 80°C on BHI agar plates. Unheated samples were plated for total number of CFUs (spores and vegetative cells).

The diarrhoeal non-haemolytic enterotoxin production was measured using the *Bacillus* Diarrhoeal Enterotoxin Visual Immunoassay kit (TECRA International Pty) following the instructions of the manufacturer. The amount of the toxin was measured in supernatants of the samples taken for RNA isolation for the microarray.

The growth at 12°C was monitored in two different ways, by cultivation in aerated conditions (similar to 30°C) described above and by streaking overnight cultures on a BHI agar plate and incubating at 12°C for up to 12 days.

### Biofilm formation

The biofilm formation by the WT, the *rpoN* mutant and the complemented strain was tested on stainless steel coupons (AISI 304, surface finish 2B) with the size of 22:18 mm, placed vertically in a 12-well plate (Cellstar, suspension culture plate, Greiner). The wells were half filled with 3 ml BHI broth and inoculated with 1.5% overnight culture. Coupons were washed and sterilized in advance as described previously [30]. Biofilms formed on the coupons were visualized by staining in 0.1% crystal violet for 30 min and subsequently washing in demineralized water to remove the excess stain.

### Motility assay and flagella staining

Swimming motility was tested on BHI plates supplemented with 0.3% agar on which 5 μl of overnight culture was spotted in the centre and incubated for 48 h at 30°C.

For flagella staining the procedure described previously [31] was followed. The cells used for flagella staining were taken from the edges of the colonies formed on the above described swimming plates.

### DNA manipulation techniques

Chromosomal DNA was isolated from *B*. *cereus* using the Wizard Genomic DNA Purification kit (Promega) for sequencing reactions and using InstaGene Matrix (Biorad) for fast colony PCR screening. Plasmid DNA was extracted using QiaPrep spin miniprep columns (Qiagen). Oligonucleotide primers (Table 1) were synthesized by Sigma. PCR amplification for cloning and sequencing was performed using KAPA HiFi Hotstart DNA Polymerase (Kapa Biosystems, Inc. Wilmington) whereas DreamTaq DNA polymerase (Fisher Scientific) was used for control PCR reactions. Restriction enzymes, T4 DNA ligase and FastAP Termosensitive Alkaline Phosphatase were obtained from Fisher Scientific and used according to manufacturer’s instructions. Plasmid constructs and *B*. *cereus* deletion and complementation clones were confirmed by DNA sequencing (Baseclear, Leiden, The Netherlands).

**Table 1:** Primers used for *rpoN* mutant construction and complementation, in 5’ to 3’ orientation. Restriction sites are **in bold**.

rpoN-1	AATCT **GAATTC** ACTGCTGTGCTTTTTAT
rpoN-2	TGCA **GCGGCCGC** CTTCAAACTAATCTCCCCCTT
rpoN-3	TCTA **GCGGCCGC** ATAGGTGAAGAAGATGAAAGTTG
rpoN-4	GGCA **GTCGAC** TCGCTACTAACATGGTCTGGAACA
BC5143compl_F	GCAT **TCTAGA** ATCCCTCTGGGCGCGTCAAAAA
BC5143compl_R	CATC **CTGCAG** AGTACAACTTTCATCTTCTTCACCTAT
BCp0019_F	GAAGGCGATGTTGTAAGAAATGTT
BCp0019_R	TCCGGTGCGTAGCGTGTT

### Deletion mutant construction and complementation

To elucidate the role of *rpoN* in *B*. *cereus* ATCC14579 an antibiotic marker-free deletion mutant, designated FM145143, was constructed using the temperature-sensitive suicide plasmid pAUL-A [32]. Flanking regions of this gene were amplified from *B*. *cereus* chromosomal DNA using primers rpoN-1 to rpoN-4 (Table 1) and purified using the MiniElute PCR purification Kit (Qiagen). The PCR products were digested and purified using a MiniElute Reaction Cleanup Kit (Qiagen). The temperature-sensitive suicide plasmid pAUL-A was digested with EcoRI and SalI followed by alkaline dephosphorylation. The treated plasmid was purified using Phenol chloroform extraction and the resulting plasmid backbone ligated with the digested flanking regions, fused in frame by introduction of a NotI site. The ligation mix was introduced into MAX Efficiency *E*.*coli* DH5α competent cells (Invitrogen) as described by the manufacturer, plated on LB containing 250 μg/ml erythromycin and obtained transformants were checked by PCR and sequencing. The resulting plasmid pAUL-Δ*rpoN* was transformed into *B*. *cereus* ATCC 14579 by electroporation (400Ω, 25 μF, 1.2 kV, 2 mM Gene Pulser Cuvette: BIORAD) and plated on BHI and grown at 30°C in the presence of 10 μg/ml erythromycin (E10). pAUL-Δ*rpoN* integration was achieved by growing the plasmid carrying strain, while shaking, for 16 hours at 42°C in a 250 ml shaking flask containing 50 ml BHI in the presence of E10. A volume of 500 μl of this overnight culture was transferred into a new shaking flask containing 50 ml BHI without antibiotics and grown overnight at 30°C, to induce double crossover events. This overnight culture was diluted and subsequently plated on BHI and grown at 37°C. Single colonies were replica plated on BHI with and without E10. PCR analyses and DNA sequencing of E10 sensitive colonies confirmed the correct 1296 bp internal in-frame deletion of *rpoN*.

Sequencing also revealed a point mutation in the *cggR* gene (BC5141) flanking the *rpoN*. The *cggR* gene encodes a repressor of five glycolytic genes downstream of *cggR* [33]. Four of those glycolytic genes (BC5135-5138) were repressed in the mutant compared to the WT during static growth and were unaffected during shaking growth. This effect was relieved in the complemented mutant and suggests that the observed phenotypes could not be ascribed to the point mutation or potential polar effect in flanking genes or other regulatory elements.

Complementation of the Δ*rpoN* deletion strain was performed by a low copy number plasmid (approximately 15 copies per cell [34]) carrying the full length *rpoN* gene including 300 bp of its upstream region. This fragment was amplified from chromosomal DNA of the WT strain using primers BC5143compl_F and BC5143compl_R (Table 1) that included a tag with recognition sites for PstI and XbaI restriction enzymes. The plasmid pHT315 [34, 35] and the insert were digested with PstI and XbaI and ligated resulting in complementation vector pHT315_BC5143compl. This complementation vector was introduced into the *rpoN* deletion strain by electroporation as described above. To maintain the plasmid, the complemented strain was pre-cultured in the presence of E10. During the experiments no antibiotic pressure was used in order to prevent secondary growth effects. Total Viable Count (TVC) plating with and without E10 did not show loss of the complementation vector. The maintenance of pBClin15 in *B*. *cereus* ATCC14579 and its derivatives was checked using plasmid specific primers BCp0019_F and BCp0019_R (Table 1).

### RNA isolation, cDNA synthesis and labelling

RNA was isolated from liquid cultures of the WT, the Δ*rpoN* and *ΔrpoN*-comp strains in BHI grown with aeration and statically. Aerated cultures were sampled at two time points, upon reaching OD (600 nm) values of 0.2 (shaking t1) and 1 (shaking t2), which corresponded to mid-exponential and end-exponential growth phases, respectively. Statically grown cultures were sampled at OD = 0.2 (600 nm) corresponding to mid-exponential growth (static). Cultures were centrifuged in 50 ml Falcon tubes for 1 min at room temperature (11.000 x g). Immediately after centrifugation the pellet was re-suspended in 1 ml TRI reagent (Ambion) by vortexing, snap frozen in liquid nitrogen and stored at -80°C until use. RNA was extracted according to the RNAwiz (Ambion) protocol. Residual DNA was enzymatically removed using the TURBO DNA-free (Ambion) kit following the instructions of the manufacturer. The quality of the extracted RNA was checked by using the Bioanalyzer (Agilent) with the Agilent RNA 6000 Nano kit, according to manufacturer’s instructions. Complementary DNA (cDNA) with amino-allyl-labelled dUTP (Ambion) was synthesized from RNA by using Superscript III reverse transcriptase (Invitrogen). Labelling and hybridization were performed as described elsewhere [36]. Two independent biological replicates were hybridized on the arrays with either 2 (WT) or 3 (Δ*rpon* and Δ*rpoN*-comp) technical replicates for each biological replicate, which were labelled with the swapped dyes (Cy3 and Cy5).

### Microarray design and data analysis

Custom-made array design for *B*. *cereus* ATCC 14579 was based on the 8 x 15K platform of Agilent Technologies (GEO accession number GPL9493, 3^rd^ design) and the genome sequence of *B*. *cereus* ATCC 14579 (NCBI accession number NC_004722). Microarrays were scanned with an Agilent G2505C scanner with two different intensities from which the optimal version was used. Image analysis and processing were performed with the Agilent Feature Extraction software (version 10.7.3.1). Transcriptome profiles were normalized using LOWESS normalization [37] as implemented in MicroPreP [38]. The data were corrected for inter-slide differences on the basis of total signal intensity per slide using Postprep [38] and median intensity of the different probes per gene was selected as the gene expression intensity. CyberT software was used to compare the different transcriptomes [39], resulting in gene expression ratios and false discovery rates (FDR) for each gene. The gene was considered significantly differentially expressed when FDR-adjusted p-value was < 0.01 and expression fold change was higher than 3 (log2 ratio>1.58 for up regulation, and <-1.58 for down regulation). To study the effect of the *rpoN* gene deletion in *B*. *cereus* the transcription profiles of the deletion mutant were compared to the WT. In order to see the effect of complementation, the complemented strain was compared to the WT. The expression of a certain gene was considered to be restored close to the WT level if the expression ratio *rpoN*-comp over WT was smaller than 3 fold and had a non-significant fdr>0.01 value. The subsets of significantly affected genes were analysed for overrepresented KEGG metabolic pathways with the web-based tool for functional analysis of genes FIVA [40]. Microarray raw and processed data is deposited in the GEO database (http://www.ncbi.nlm.gov/geo/) under accession number GSE65894.

### Statistical analysis

All described experiments were performed in independent biological triplicates, unless stated otherwise. Presented data in the graphs are the averages of the replicates ± the standard deviation. Differences were considered statistically significant if the p value from student t-test (MS Excel2010) for two samples with equal variance was p<0.05. For the microarray gene expression ratios were considered relevant when the fdr<0.01.

## Results

### Deletion of *rpoN* affects the colony morphotype and cell morphology of *B*. *cereus* ATCC 14579

**Fig 1:**
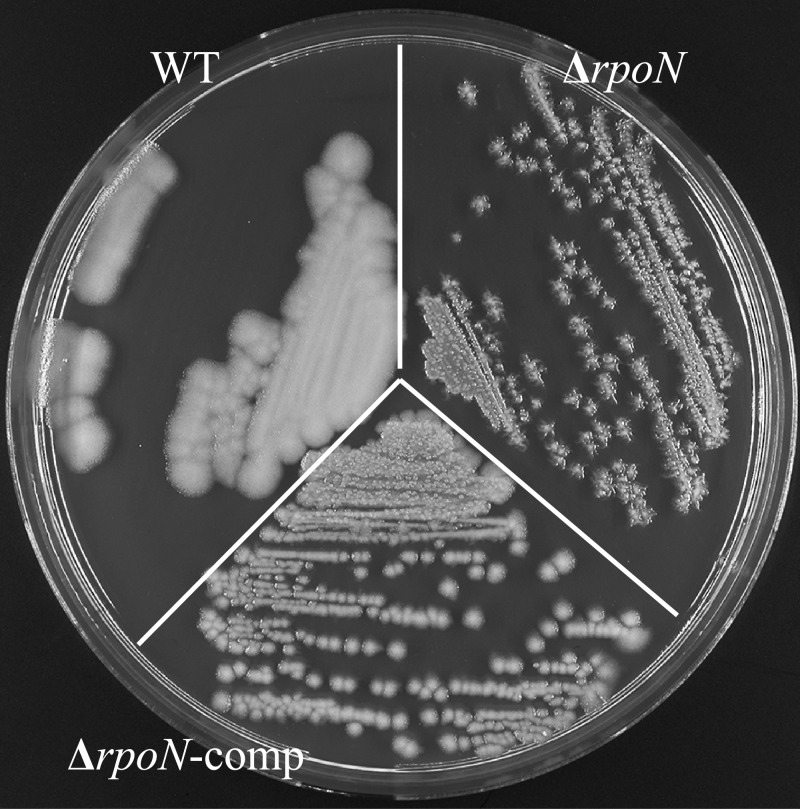
Colony morphotypes. Colonies of *B*. *cereus* ATCC 14579 WT (a), its Δ*rpoN* mutant derivative (b) and the complemented mutant (Δ*rpoN*-comp) (c) cultivated on BHI plates for 24 h at 30°C.

**Fig 2:**
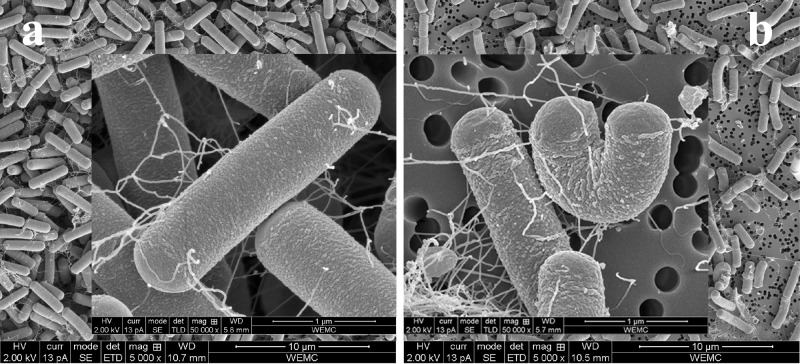
Cells of the WT and its Δ*rpoN* mutant. SEM images of aerobically grown overnight cultures of *B*. *cereus* ATCC 14579 WT (a) and its Δ*rpoN* mutant derivative (b) in BHI at 30°C.

*B*. *cereus* ATCC 14579, its *rpoN* mutant and complemented strain were routinely streaked on BHI plates with 1.5% agar. On this medium the *rpoN* mutant displayed a characteristic dendritic (branched) colony morphology (Fig 1) in contrast to the round shaped and wide-spread colonies of the wild type strain (WT). The colony morphotype was partially restored in the complemented *rpoN* mutant albeit that the colony size remained reduced compared to the WT. Images of the *rpoN* mutant and WT cells from aerated overnight cultures obtained by Scanning Electron Microscopy (SEM) revealed differences in the morphology of single cells (Fig 2), with the mutant population displaying relatively more curved cells (~7% versus <0.7% for the WT) with a less smooth cell surface compared to the WT.

### The *rpoN* gene is essential for growth under oxygen limitation and at low temperature

**Fig 3:**
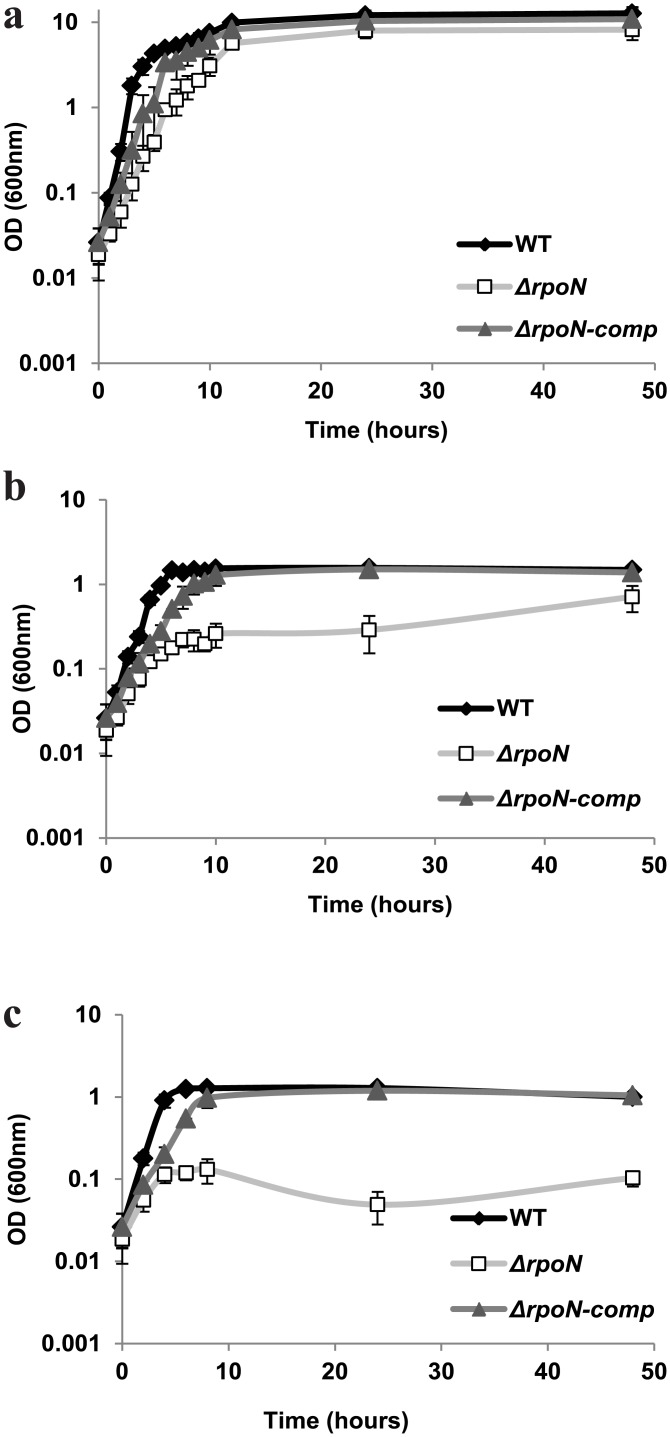
Growth. Growth of *B*. *cereus* ATCC 14579 WT, Δ*rpoN* and Δ*rpoN*-comp in BHI in three conditions with different oxygen availability a) aerated, b) static and c) anaerobic at 30°C.

**Fig 4:**
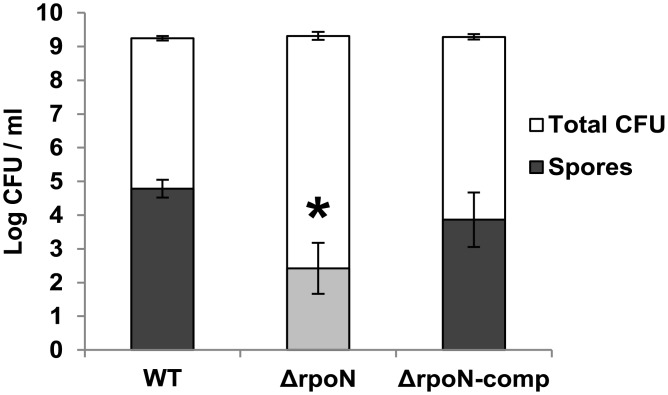
Spore formation. Number of spores and total viable counts in BHI formed by *B*. *cereus* ATCC 14579 WT, Δ*rpoN* and Δ*rpoN*-comp in BHI following aerated growth at 30°C for 48 h. The asterisk indicates significant difference (student’s t test, p<0.05) of the Δ*rpoN* compared to both the WT and Δ*rpoN*-comp.

The growth of the WT, its *rpoN* mutant derivative (Δ*rpoN*) and complemented *rpoN* mutant (*rpoN*-comp) was measured under aerated (with shaking), static and anaerobic conditions (with shaking). The static and anaerobic growth of the mutant was drastically impaired compared to the WT as determined by measurement of optical density at 600 nm (Fig 3). The aerobic growth was affected to a lesser extent. For all conditions tested, the phenotype of the *rpoN* mutant was restored to WT behaviour upon complementation with a plasmid-encoded copy of the *rpoN* gene. In addition, spore numbers in BHI after 48 h of aerated growth were significantly reduced for the *rpoN* mutant in comparison with the WT and the complemented strain (Fig 4).

**Fig 5:**
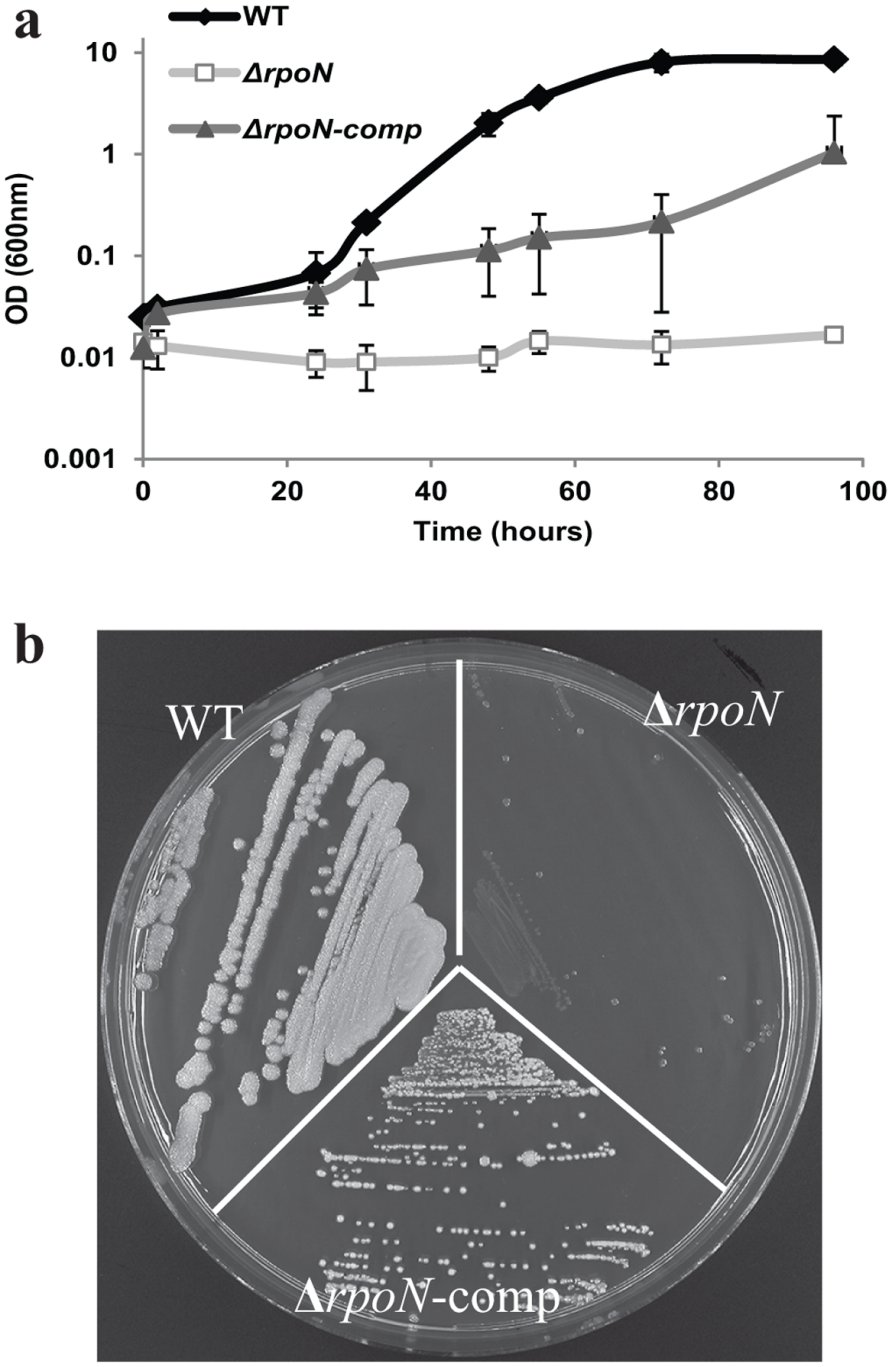
Growth at low temperature. Growth of *B*. *cereus* ATCC 14579 WT, Δ*rpoN* and Δ*rpoN*-comp at 12°C. a) Aerated growth in BHI monitored up to 96 h, b) growth on BHI agar plate, picture was taken on day 12.

The *rpoN* mutant was unable to grow at 12°C with aeration in BHI broth over a period of 4 days during which the OD at 600 nm was measured (Fig 5a). Upon prolonged incubation on BHI agar plates the colonies of the mutant were smaller and almost invisible compared to the WT and complemented strain when grown for up to 12 days at 12°C (Fig 5b).

### Carbohydrate metabolism

The ability of the WT, *rpoN* mutant and its complemented counterpart to ferment different carbohydrates was studied using the API CH50 test (BioMerieux). The *rpoN* mutant was able to ferment most of the sugars that were metabolized by the parental strain, with the exception of arbutin, esculin and cellobiose. These carbohydrates share a common structure, the first two are glucosides, and cellobiose is a disaccharide of glucose. All three carbohydrates share the same structural features as the building blocks of chitobiose, which is a glucosamine dimer.

### Sigma 54 is essential for flagella biosynthesis and affects biofilm formation and toxin production

**Fig 6:**
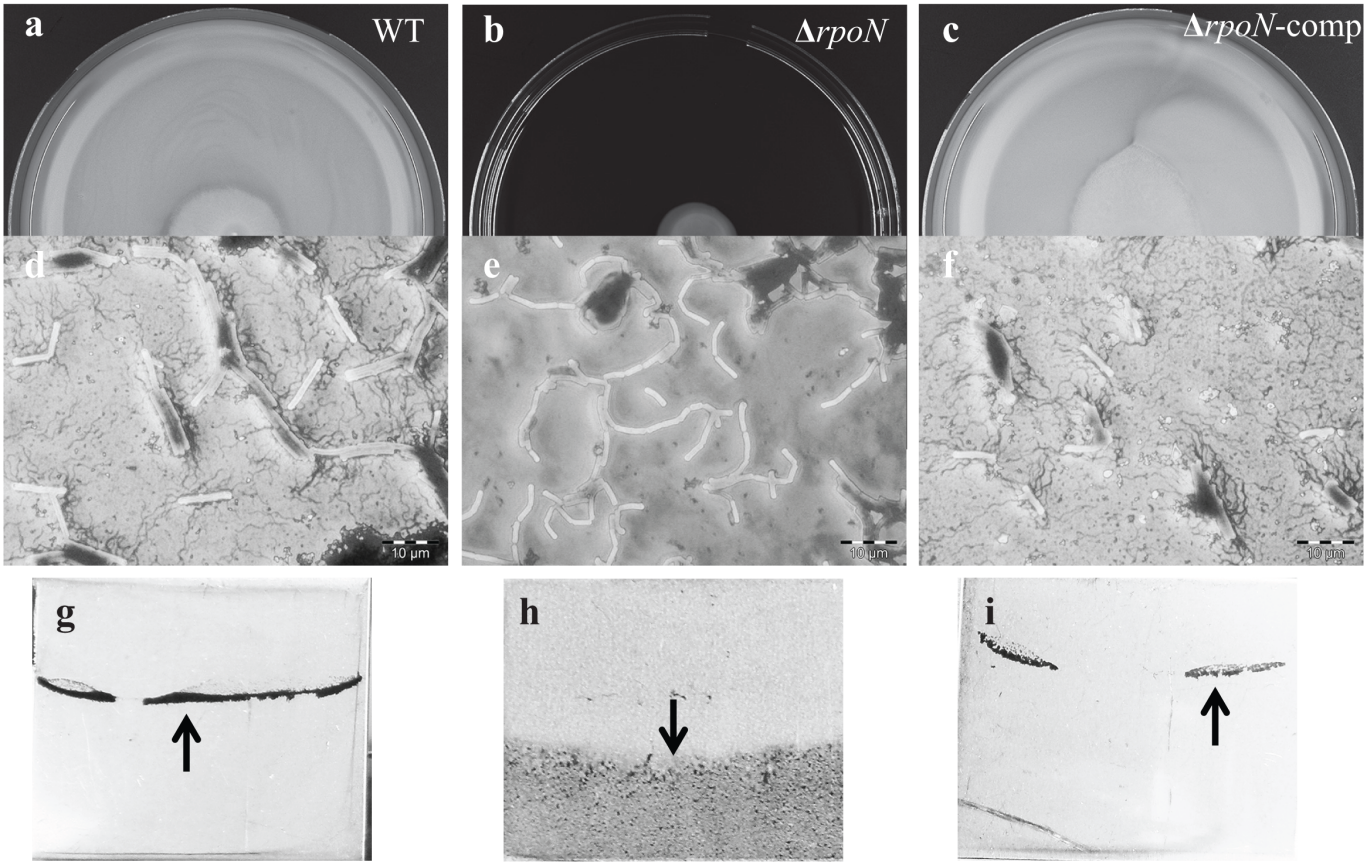
Motility and biofilm formation. Motility, presence of flagella and biofilm formation by *B*. *cereus* ATCC 14579 WT, Δ*rpoN* and Δ*rpoN*-comp. (a-c) Motility on swimming BHI plates with 0.3% agar after 48 h, (d-f) flagella staining of cells taken from the swimming plate, (g-i) Static biofilm formation on stainless steel coupons partly submerged in BHI after 48 h at 30°C. Biofilms were stained with crystal violet.

The swimming motility of the WT, *rpoN* mutant and the complemented strain was tested on BHI plates with 0.3% agar (Fig 6a, 6b and 6c) and showed that the *rpoN* mutant was severely impaired in motility (Fig 6a). Further support for the lack of motility of the *rpoN* mutant was obtained by flagella staining (Fig 6d, 6e and 6f). Notably, flagella could not be visualized for the mutant cells taken from the colony on swimming agar surface (Fig 6e), in contrast to the hyperflagellated cells of the WT and *rpoN*-comp (Fig 6d and 6f).

*B*. *cereus*, including strain ATCC 14579 typically forms biofilms attached to polystyrene or stainless steel surfaces at the air-liquid interface [41, 42]. The *rpoN* mutant lost the ability to form robust biofilms of this type, but instead formed a submerged biofilm on the surface of the stainless steel coupons with lower intensity of staining (Fig 6h).

**Fig 7:**
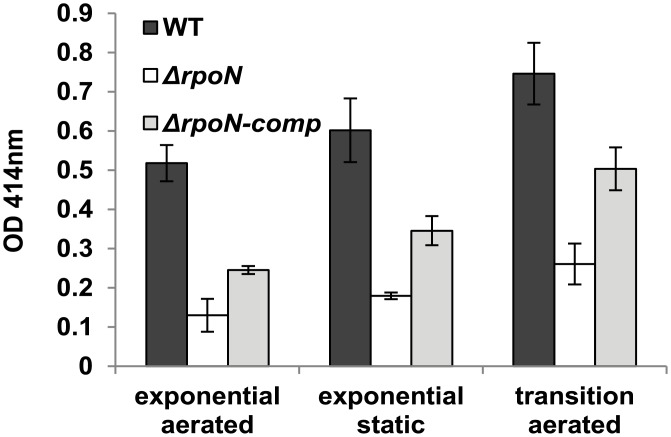
Toxin production. Production of non-hemolytic toxin (NheA) measured in BHI at 30°C with aeration at mid-exponential (OD 600 nm = 0.2) and end-exponential (OD 600 nm = 1) growth phases, and at mid-exponential (OD 600 nm = 0.2) phase during static growth.

Production of non-haemolytic enterotoxin lytic component L2 (NheA) was measured in aerated (mid-exponential and end-exponential) and static (mid-exponential) growing conditions. Toxin levels for the *rpoN* mutant were significantly lower compared to the WT, with ratios of WT/Δ*rpoN* levels varying between 2.9 and 4 depending on the condition tested (Fig 7). The toxin level was partially restored to that of the WT for the complemented mutant strain.

### Altered gene expression profile upon deletion of the *rpoN* gene

Gene expression profiles of the WT, the *rpoN* mutant and the complemented mutant were investigated for aerobically grown cultures at mid-exponential and end-exponential growth phases, and for static cultures at mid-exponential phase. The profiles of the WT and *ΔrpoN*-comp clustered together and separately from *ΔrpoN* (S1 Fig). This confirms that the observed effects of *rpoN* deletion on gene expression result mainly from deletion of the target gene and not from possible polar effects on neighbouring genes or regulatory elements.

The number of genes significantly affected in the *rpoN* mutant was the lowest in mid-exponentially (shaking t1) growing cells with aeration (598, from which 118 were down and 480 up regulated in the mutant). 39% of the affected genes were restored to WT level by complementation. In the end-exponential phase (shaking t2) with aeration 993 genes were altered in the mutant, of which 320 were down and 673 up regulated. Static growth affected the *rpoN* mutant most dramatically, with 1535 (854 up and 681 down regulated) gene expressions significantly changed compared to the WT. In the complemented mutant, gene expression was restored to WT levels by 48% (shaking t2) and 78% (static).

**Fig 8:**
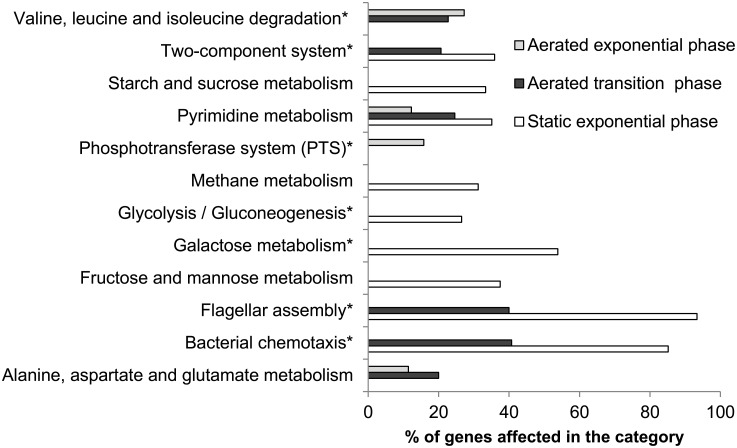
Affected metabolic pathways. Overrepresented metabolic pathways significantly down regulated in Δ*rpoN*. Outcome of FIVA analysis of significantly affected genes. Categories with asterisk (*) were restored to close to WT condition by complementation.

**Fig 9:**
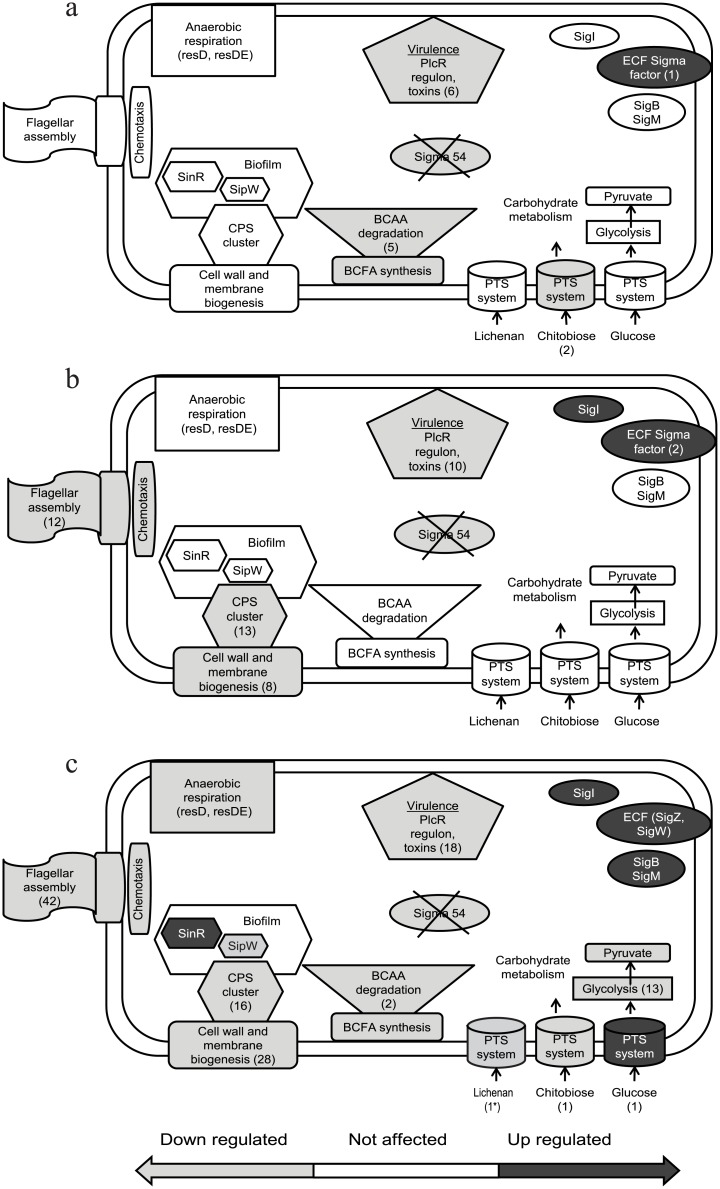
Affected processes in the cell. Schematic model of cellular processes, based on observed phenotypes combined with affected and complemented genes in the *rpoN* mutant. a) Aerated growth mid-exponential phase, b) aerated growth end-exponential phase, c) static growth mid-exponential phase. The numbers in (brackets) are the number of genes affected in the given category. Asterisk (*) means the gene was not complemented.

For the transcriptome data analysis our initial focus was on functions lost in the *rpoN* mutant but restored to near WT levels by complementation. The KEGG Metabolic pathways were analysed for functionalities down regulated and overrepresented in the mutant (Fig 8). Most dominant categories identified refer to flagellar assembly, two component systems, aminoacid metabolism, carbohydrate metabolism and phosphotransferase systems (PTS). The affected cellular processes in the *rpoN* mutant are schematically presented in Fig 9a, 9b and 9c for mid-exponential aerated, end-exponential aerated and exponential static growth phases respectively.

### Transcriptome responses linked to phenotypes and prediction of the Sigma 54 regulon

Specific transcriptome responses showed good agreement with the observed phenotypes such as cell wall biogenesis, carbohydrate metabolism, toxin production, motility and biofilm formation. In relation to cell surface morphology, transcriptome analysis showed several (14 in aerated condition at end-exponential phase, 36 under static condition) membrane and cell wall biogenesis related genes down regulated in the mutant (Table A in S1 File). As support for observed phenotypes related to carbohydrate utilisation (APICH50 test) in the *rpoN* mutant, BC0807 encoding a component of the diacetylchitobiose-specific sugar transporting PTS system was downregulated in the mutant, along with BC5211 encoding lichenan oligosaccharide specific PTS component in shaking t1 and static conditions (Table B in S1 File). The latter belongs to the orthologous group of cellobiose-specific PTS systems. A Sigma 54 promoter binding site was identified upstream BC5211 as a result of an *in silico* search using the -12–24 consensus promoter region by Francke et al. [23] (Table C in S1 File, previously unpublished). Furthermore the gene encoding for the EBP *levR* (BC5205) is located in proximity, which suggests that this PTS system is most likely regulated by Sigma 54. In addition, several genes enconding enzymes, such as chitooligosaccharide deacetylase (BC0171) and 6-phospho-beta-glucosidase (BC5209), involved in initial stages of utilisation of the above mentioned carbon sources were also down regulated in the mutant (Table B in S1 File).

The role of Sigma 54 in cold shock survival of *B*. *subtilis* has been linked previously to synthesis of branched chain fatty acids which can be formed by degradation of branched amino acids, such as isoleucine [17]. Enzymes encoded by the *bkd* operon are involved in this conversion. Similar to *B*. *subtilis* [18], the gene encoding the BkdR regulator (BC4165) is located in the flanking region of the *B*. *cereus bkd* operon (BC4163-4157) which was down regulated in the *rpoN* mutant in all three conditions tested (Table D in S1 File). Furthermore, a Sigma 54 promoter binding site was identified upstream of BC4163 (Table C in S1 File), which suggests that the *bkd* operon is regulated by Sigma factor 54 in *B*. *cereus*. Reduced expression of the *bkd* operon could explain the impaired growth of the *rpoN* mutant observed at low temperature.

Loss of motility was accompanied by lower expression levels of genes involved in flagellar biosynthesis and chemotaxis, including the gene that encodes FlhF, a putative transcriptional regulator [43], in the mutant compared to the WT at shaking t2 (12 genes) and especially in static conditions (42 genes) (Table E in S1 File).

The transcriptome data revealed up regulation of SinR, a known negative regulator of biofilm formation in *B*. *subtilis* [44], in the *rpoN* mutant in shaking t1 and static conditions, which was restored by complementation in static condition. Interestingly, even though SinI, the antagonist of SinR, was also up regulated in the mutant in all conditions (Table F in S1 File), *sipW* encoding a signal peptidase, which processes the EPS component TasA and is important for *B*. *subtilis* and *B*. *cereus* biofilm formation [45], was down regulated in static condition. Furthermore, genes involved in putative capsular polysaccharide (CPS) biosynthesis (BC5263-BC5278 [46]) were down regulated in the mutant and reverted to WT levels in static (16 genes) and shaking t2 (13 genes) conditions upon complementation (Table F in S1 File).

The *gab* gene cluster, involved in γ-aminobutyric acid (GABA) shunt, as well as the lysine-2,3-aminomutase gene are regulated by Sigma 54 in *B*. *thuringiensis* [20, 21, 47]. GabT (BC0355) and Lysine 2.3-aminomutase (BC2251) were predicted to be in the regulon of Sigma 54 in *B*. *cereus* (this study, Table C in S1 File). Both of these genes are located in the proximity of Sigma 54 EBPs (BC0356 and BC2250 respectively, for the full list of EBPs refer to supplement 2 in [23]). However, the expression of these two genes was not affected in the *rpoN* mutant.

Lower non-haemolytic enterotoxin (NHE) production by the *rpoN* mutant was supported by lower expression of the NHE-lytic component L2 gene (BC1809, Table G in S1 File). The expression of several other predicted or confirmed virulence factors [48], including the virulence regulator PlcR were also down regulated in the mutant, for the affected genes (6 in shaking t1, 10 in shaking t2, 18 in static) see Table G in S1 File.

Differential expression of sporulation related genes such as AbrB (BC0042) and Stage V sporulation protein S (BC2142) (Table I in S1 File) was observed in end-exponential phase cells, which may have affected sporulation efficiency in the mutant, but more studies are required to support a direct role for Sigma 54 in sporulation control.

## Discussion

Even though deletion of the *rpoN* gene was not lethal to *B*. *cereus* ATCC 14579, a wide range of cellular functions was affected as reflected both in phenotypic response and in gene expression patterns of the *rpoN* mutant compared to the WT. Phenotypes that were affected in the *rpoN* mutant included growth under anaerobic conditions and at low temperature, biofilm formation and motility, sporulation and toxin production. Most of these functions are part of survival strategies of *B*. *cereus*, in line with described roles of Sigma 54 in adaptation to unfavourable environmental conditions for *Listeria monocytogenes* and *Escherichia coli* [9, 49]. A common theme for Sigma 54 among different species can be found in regulation of processes related to flagellar biosynthesis and motility. Its role in motility has been previously reported for different microorganisms, such as *Campylobacter jejuni* [50], *L*. *monocytogenes* [49], *Vibrio fischeri* [10], *E*. *coli* [11] and *Pseudomonas fluorescens* [29]. In addition, a multispecies *in silico* study pointed at a strong positive correlation between the presence of the Sigma 54 encoding gene and the ability of the species to form flagella [23]. However, motility of a *B*. *subtilis rpoN* mutant was not affected [5]. In our study the ability to synthesize flagella was lost in the *rpoN* mutant of *B*. *cereus* ATCC 14579, shown both by phenotypes and flagellar gene expression, thus supporting the role of Sigma 54 in motility in this species.

The role of Sigma 54 in motility in literature is often intertwined with its role in biofilm formation [10, 16, 51], which is not surprising since for a wide range of microorganisms, motility was found to be a prerequisite for biofilm formation [52, 53]. Biofilm formation was reduced upon deletion of the gene encoding Sigma 54 for various species, including *Vibrio anguillarum* [16] and *Burkholderia cenocepacia* [51]. By contrast, the *rpoN* mutant of *Enterococcus faecalis* was resistant to autolysis and formed more robust biofilms conceivably due to altered relative composition of extracellular components [12]. *V*. *fischeri* produced a more widespread biofilm with less intense CV staining compared to the WT upon deletion of the *rpoN* gene. This effect could not be ascribed to the loss of motility since a flagella-less mutant of *V*. *fischeri* could still form WT-like biofilms [10]. In our study, motility loss could be responsible for the appearance of a submerged biofilm of the *B*. *cereus rpoN* mutant, while both the WT and complemented mutant strain formed only air-liquid biofilms. This is in line with another study showing importance of motility for formation of air-liquid interface biofilms [52]. In addition to motility loss, other factors may have contributed to reduced biofilm formation in the *rpoN* mutant, including downregulation of *sipW* and the putative CPS cluster encoding capsular polysaccharide biosynthesis genes (Table F in S1 File), and slower growth in static conditions.

The *rpoN* mutant formed very distinctive dendritic colonies on BHI plates (Fig 1). This type of colony morphology has been described previously for a PlcR deletion mutant of *B*. *cereus* ATCC 14579 albeit on nutrient poor swarming agar. This phenotype was caused by overproduction of a biosurfactant leading to sliding behaviour of the non-flagellated mutant [54]. It was suggested that the production of the biosurfactant is negatively regulated by PlcR. Notably, the expression of PlcR and its regulon members was downregulated in the *rpoN* mutant (Table G in S1 File), but it remains to be determined whether the dendritic colony formation is caused by altered surfactant production.

PTS systems are an important part of carbon metabolism in bacteria since they catalyse the transport of sugars and their derivatives. Some PTS systems have been shown to be under the control of Sigma 54 in different microorganisms [15, 55, 56]. Around 3% of all predicted EBPs involved in Sigma 54 mediated regulation have been found to be directly linked to signalling via PTS systems [23]. In addition to mediating nutrient uptake, PTS systems play a regulatory role in metabolism [56, 57] and a role in biofilm formation by *B*. *cereus* [58] has been described. In our study the expression of diacetylchitobiose-specific and sucrose-specific PTS systems was down regulated in the *rpoN* mutant (Table B in S1 File) showing the positive regulation of those PTS systems by Sigma 54.

The growth of the *rpoN* mutant was significantly impaired in static and anaerobic conditions, which indicates that the role of Sigma 54 is more pronounced in environments with oxygen limitation. Similarly, a *Campylobacter jejuni rpoN* mutant showed survival defects in static cultures compared to those grown with aeration [13]. A possible link for impaired static growth of the *rpoN* mutant of *B*. *cereus* could be down-regulation of the two component regulatory genes *resD* and *resE* required for anaerobic respiration in *B*. *subtilis* [59] and respiratory nitrate (nar) and nitrite (nas) reductase operons [26] (Table H in S1 File). On the other hand, *B*. *cereus* is able to switch to fermentative metabolism under anaerobic conditions, however for this pyruvate availability is important [25], and this intermediate is produced via glycolysis in the cell, a pathway which was downregulated in the *rpoN* mutant during static growth.

An *in silico* search by Francke et al. [23] (data presented in Table C in S1 File, previously unpublished) based on the conserved promoter binding sites of Sigma 54 found putative binding sites in proximity of 32 genes in *B*. *cereus* ATCC 14579 (Table C in S1 File). A similar search by Peng et al. (2015) in *B*. *thuringiensis* revealed 16 positions, from which 9 gene functions are overlapping with our predicted regulon. In our transcriptome study, 16 out of those 32 genes were significantly affected (fdr<0.01, without using any cut off for expression) in the *rpoN* mutant at least in one of the conditions. Seven of those genes were under positive regulation by Sigma 54, and included genes involved in cell metabolism such as phosphate butyryltransferase (BC4163), PTS system lichenan-specific IIC component (BC5211), methionine aminopeptidase (BC0153), undecaprenyl pyrophosphate phosphatase (BC0677) and 6-phosphogluconate dehydrogenase-like protein (BC2225). However, several of the other predicted regulon members were significantly upregulated in the *rpoN* mutant, such as MarR family transcriptional regulator (BC2434) and PhaR protein (BC1316), suggesting the existence of additional regulatory networks or indirect regulation. The other 16 genes were not significantly affected, which may be due to specificity of growth conditions required for expression of these genes.

Several phenotypes such as the toxin production and low temperature growth were only partly restored, which could be due to the fact that the re-introduction of the *rpoN* gene on a plasmid is not identical to the WT situation. Such incomplete complementation of the *rpoN* has been reported previously for *L*. *monocytogenes* [60] and *E*. *faecalis* [12].

In all conditions the number of genes up regulated in the *rpoN* deletion mutant exceeded the number of down regulated genes. Sigma 54 is known to act as an activator and not a repressor of gene expression [61], which seems in contradiction with our data. However, it may suggest that up regulation of genes in the *rpoN* mutant results from indirect effects of *rpoN* deletion, for example via other affected regulators. Indeed, regulators such as several extra-cytoplasmic function (ECF) sigma factors and Sigma B (in static growth) were affected in the *rpoN* mutant (Table I in S1 File), or *codY* [62] which was down regulated in static growth. Furthermore, due to the link of Sigma 54 to metabolism and environmental response, the effects of *rpoN* deletion can also be medium or temperature dependent, which was already shown for motility of an *E*. *coli rpoN* mutant [11].

To conclude, this study provides experimental evidence that in *B*. *cereus* Sigma 54 is involved either directly or indirectly in a large range of cellular processes such as growth at low temperature and in anaerobic conditions, motility and biofilm formation and toxin production. All observed phenotypes indicate that Sigma 54 of *B*. *cereus* regulates metabolic rearrangements as a survival strategy to adapt to different environmental niches, ranging from soil, via food processing environments and foods, to human.

## Supporting Information

S1 Fig Clustering of WT, Δ*rpoN* mutant and Δ*rpoN*-comp. Clustering of array samples based on expression patterns (example on aerated end-exponential phase). Data was normalized per gene and per row and clustered using UPGMA & pearson correlation in Genesis [63].
(EPS)

S1 File Transcriptomic data. Relevant gene expression ratios in the *rpoN* mutant compared to WT. Genes are grouped in tables by their functions. **Table A in** S1 File. **Cell wall and membrane biogenesis**. Genes related to cell wall and membrane biogenesis significantly down regulated in the *rpoN* mutant (p<0.01, ratio >3). Genes that are also a part of the CPS cluster in Biofilm related table are not included here. **Table B in** S1 File. **Carbohydrate metabolism**. Genes related to carbohydrate metabolism significantly down regulated in the *rpoN* mutant (p<0.01, ratio >3). **Table C in** S1 File. **Predicted Sigma 54 regulon**. Predicted regulon members of Sigma 54 according significantly affected in the *rpoN* mutant in the transcriptomic study (p<0.01, no cutoff for expression ratio). The predicted regulon members were obtained as described in [23], by in silico search of the conserved -12-24 promoter region of Sigma 54. **Table D in** S1 File. **Aminoacid metabolism**. Genes involved in Valine, leucine and Isoleucine degradation significantly down regulated in the *rpoN* mutant (p<0.01, ratio >3). **Table E in** S1 File. **Motility**. Genes related to motility significantly down regulated in the *rpoN* mutant (p<0.01, ratio >3). **Table F in** S1 File. **Biofilm formation**. Genes related to biofilm formation significantly affected in the *rpoN* mutant (p<0.01, ratio >3). **Table G in** S1 File. **Virulence**. Genes related to virulence significantly affected in the *rpoN* mutant (p<0.01, ratio >3). **Table H in** S1 File. **Anaerobic respiration**. Genes relevant for anaerobic respiration significantly down regulated in the *rpoN* mutant (p<0.01, ratio >3). **Table I in** S1 File. **Regulators and sporulation related genes**. Sigma factors significantly affected in the *rpoN* mutant (p<0.01, ratio >3).
(DOCX)
