# Supplementary material for: Bacillus cereus ATCC 14579 RpoN (Sigma 54) Is a Pleiotropic Regulator of Growth, Carbohydrate Metabolism, Motility, Biofilm Formation and Toxin Production
Source: PLoS One. 2015 Aug 4;10(8):e0134872. doi: 10.1371/journal.pone.0134872 (PMC4524646; doi:10.1371/journal.pone.0134872)
Supplement: S1 File — Relevant gene expression ratios in the rpoN mutant compared to WT. Genes are grouped in tables by their functions. Table A in S1 File. Cell wall and membrane biogenesis. Genes related to cell wall and membrane biogenesis significantly down regulated in the rpoN mutant (p<0.01, ratio >3). Genes that are also a part of the CPS cluster in Biofilm related table are not included here. Table B in S1 File. Carbohydrate metabolism. Genes related to carbohydrate metabolism significantly down regulated in the rpoN mutant (p<0.01, ratio >3). Table C in S1 File. Predicted Sigma 54 regulon. Predicted regulon members of Sigma 54 according significantly affected in the rpoN mutant in the transcriptomic study (p<0.01, no cutoff for expression ratio). The predicted regulon members were obtained as described in [23], by in silico search of the conserved -12-24 promoter region of Sigma 54. Table D in S1 File. Aminoacid metabolism. Genes involved in Valine, leucine and Isoleucine degradation significantly down regulated in the rpoN mutant (p<0.01, ratio >3). Table E in S1 File. Motility. Genes related to motility significantly down regulated in the rpoN mutant (p<0.01, ratio >3). Table F in S1 File. Biofilm formation. Genes related to biofilm formation significantly affected in the rpoN mutant (p<0.01, ratio >3). Table G in S1 File. Virulence. Genes related to virulence significantly affected in the rpoN mutant (p<0.01, ratio >3). Table H in S1 File. Anaerobic respiration. Genes relevant for anaerobic respiration significantly down regulated in the rpoN mutant (p<0.01, ratio >3). Table I in S1 File. Regulators and sporulation related genes. Sigma factors significantly affected in the rpoN mutant (p<0.01, ratio >3). (DOCX) [file pone.0134872.s002.docx]

**Table A in S1 File. Cell wall and membrane biogenesis.** Genes related to cell wall and membrane biogenesis significantly down regulated in the *rpoN* mutant (p<0.01, ratio >3). Genes that are also a part of the CPS cluster in Biofilm related table are not included here.

|  |  |  |  |  | **log2 Δ*rpoN*/WT** | | |
| --- | --- | --- | --- | --- | --- | --- | --- |
| **Biological function** | **Gene** | **COG** | **Name** | **Annotation** | **shaking t1 (mid-exp.)** | **shaking t2 (end-exp.)** | **static exp.** |
| Cell wall and membrane biogenesis (COG category) | BC0054 | M | gcaD | Glucosamine-1-phosphate acetyltransferase | - | - | **-2.13** |
|  | BC0190 | M | glmS | Glucosamine--fructose-6-phosphate aminotransferase [isomerizing] | - | - | **-1.78** |
|  | BC0258 | M | murF | UDP-N-acetylmuramoylalanyl-D-glutamyl-2,6-diaminopimelate--D-alanyl-D- alanyl ligase | - | - | **-1.73** |
|  | BC0740 | M, T |  | Cell wall endopeptidase, family M23/M37 | - | - | **-1.70** |
|  | BC0887 | M |  | Collagen adhesion protein | - | - | **-3.89** |
|  | BC0888 | M | cwlH | N-acetylmuramoyl-L-alanine amidase | - | -2.76 | **-4.07** |
|  | BC0902 | M |  | S-layer protein / N-acetylmuramoyl-L-alanine amidase | -1.98 | -4.43 | -4.69 |
|  | BC0966 | M |  | Fimbria associated protein | -2.21 | -2.36 | -3.63 |
|  | BC1067 | M | pbpF | Multimodular transpeptidase-transglycosylase PBP 2C | - | - | **-2.12** |
|  | BC1277 | M |  | D-alanyl-D-alanine carboxypeptidase | - | - | **-1.88** |
|  | BC1535 | M | ypjH | Glycosyltransferase | - | - | **-1.67** |
|  | BC1660 | M | yjbJ | Soluble lytic murein transglycosylase | - | - | **-2.47** |
|  | BC2850 | MR | ykfB | Mandelate racemase/muconate lactonizing enzyme family protein | - | **-1.80** | **-2.61** |
|  | BC3090 | M | yvdQ | hypothetical protein | - | - | **-1.61** |
|  | BC3257 | T, M |  | N-acetylmuramoyl-L-alanine amidase | - | - | **-1.97** |
|  | BC3307 | M |  | D-alanyl-D-alanine carboxypeptidase | - | - | **-2.05** |
|  | BC3308 | M |  | Capsule biosynthesis protein capA | - | - | -2.10 |
|  | BC3909 | M | murB | UDP-N-acetylenolpyruvoylglucosamine reductase | - | - | **-1.63** |
|  | BC3912 | M | murD | UDP-N-acetylmuramoylalanine--D-glutamate ligase | - | - | **-1.82** |
|  | BC3913 | M | mraY | Phospho-N-acetylmuramoyl-pentapeptide-transferase | - | - | **-2.17** |
|  | BC3914 | M | murE | UDP-N-acetylmuramoylalanyl-D-glutamate--2,6-diaminopimelate ligase | - | - | **-2.05** |
|  | BC4669 | M | mscL | Large-conductance mechanosensitive channel | - | - | **-1.69** |
|  | BC4897 | M |  | IG hypothetical 16740 | - | - | **-3.68** |
|  | BC5005 | M |  | D-alanyl-D-alanine carboxypeptidase | - | -2.39 | - |
|  | BC5125 | M |  | hypothetical protein | - | - | **-2.94** |
|  | BC5237 | M | ydaM | N-acetylglucosaminyltransferase | - | **-1.74** | **-3.88** |
|  | BC5238 | M | opuD | Glycine betaine transporter | -2.02 | **-1.71** | - |
| Cell wall and membrane biogenesis (COG category) | BC5283 | M |  | Stage II sporulation protein Q | - | - | **-1.79** |
|  | BC5357 | M |  | Collagen adhesion protein | - | **-2.33** | **-4.02** |
|  | BC5358 | M |  | Collagen adhesion protein | - | **-1.87** | **-3.64** |
|  | BC5389 | M |  | D-alanyl-D-alanine carboxypeptidase | - | -2.85 | -2.37 |
|  | BC5390 | M | cwlJ | Cell wall hydrolase cwlJ | - | - | **-1.98** |
|  | BC5427 | M |  | Glycosyltransferase involved in cell wall biogenesis | - | - | **-2.08** |
|  | BC5438 | M | ysbB | Murein hydrolase export regulator | - | **-4.40** | 4.25 |
|  | BC5443 | M |  | Glycine betaine transporter | - | **-1.59** | **-3.45** |

(-) means the gene was not significantly and highly affected in the given condition.

Genes with ratios in **bold** were restored to close to WT levels by complementation.

**Table B in S1 File. Carbohydrate metabolism.** Genes related to carbohydrate metabolism significantly down regulated in the *rpoN* mutant (p<0.01, ratio >3).

|  |  |  |  |  | **log2 Δ*rpoN*/WT** | | |
| --- | --- | --- | --- | --- | --- | --- | --- |
| **Biological function** | **Gene** | **COG** | **Name** | **Annotation** | **shaking t1 (mid-exp.)** | **shaking t2 (end-exp.)** | **static exp.** |
| Carbohydrate metabolism (COG category) | BC0171 | G | ybaN | Chitooligosaccharide deacetylase | - | **-3.12** | **-3.57** |
|  | BC0202 | GEPR | yitZ | Multidrug resistance protein B | - | **-1.84** | **-3.50** |
|  | BC0219 | G |  | Glucose uptake protein homolog | - | -2.73 | **-2.80** |
|  | BC0398 | GEPR |  | Benzoate transport protein | - | - | **-2.95** |
|  | BC0413 | G | yugT | Exo-alpha-1,4-glucosidase | - | - | **-2.78** |
|  | BC0569 | G | yesQ | SN-glycerol-3-phosphate transport system permease protein ugpE | - | - | **-1.95** |
|  | BC0660 | G | rbsK | Ribokinase | - | - | **-1.86** |
|  | BC0757 | GEPR | yvmA | Bicyclomycin resistance protein | - | - | **-3.39** |
|  | BC0773 | G | ydjE | Fructokinase | - | - | -2.15 |
|  | BC0774 | G | sacA | Sucrose-6-phosphate hydrolase | - | - | -3.17 |
|  | BC0775 | G, G | sacP | PTS system, sucrose-specific IIBC component | - | **-4.18** | -3.79 |
|  | BC0807 | G |  | PTS system, diacetylchitobiose-specific IIA component | **-2.23** | - | **-1.68** |
|  | BC0896 | G |  | S-layer protein / Peptidoglycan endo-beta-N-acetylglucosaminidase | - | -2.78 | -4.51 |
|  | BC0962 | GEPR | lmrB | Lincomycin resistance protein | -1.67 | - | - |
|  | BC0981 | G |  | Dihydroxyacetone kinase | **-2.61** | - | -1.99 |
|  | BC1598 | G |  | LACX protein | - | - | **-2.26** |
|  | BC1759 | GEPR | ydeG | Tetracycline resistance protein | - | - | **-1.70** |
|  | BC2104 | G |  | hypothetical protein | - | - | **-2.07** |
|  | BC2300 | GEPR |  | Oxalate/formate antiporter | - | **-2.21** | 3.39 |
|  | BC2325 | GEPR | ykuC | Macrolide-efflux protein | - | **-1.83** | **-4.65** |
|  | BC2393 | G |  | PTS system, diacetylchitobiose-specific IIB component | **-2.36** | - | - |
|  | BC2464 | G |  | S-layer protein / Peptidoglycan endo-beta-N-acetylglucosaminidase | - | - | **-2.14** |
|  | BC3682 | G |  | Transketolase | - | - | **-1.67** |
|  | BC3718 | G, G, GT | fruA | PTS system, fructose-specific IIABC component | -2.13 | - | - |
|  | BC3719 | G | fruK | 1-phosphofructokinase | -2.70 | - | -2.35 |
|  | BC3720 | KG | fruR | Fructose repressor | - | - | **-3.28** |
|  | BC3804 | G | ylxY | Chitooligosaccharide deacetylase | - | -2.23 | **-3.05** |
|  | BC4271 | GEPR | yqgE | Transporter, MFS superfamily | - | - | **-2.03** |
|  | BC4507 | G |  | Aquaporin | - | -2.15 | -3.47 |
|  | BC4707 | GEPR |  | Multidrug resistance protein B | - | - | **-1.78** |
|  | BC4716 | G | glcU | Glucose uptake protein | - | - | **-2.12** |
|  | BC4738 | GEPR | yttB | Multidrug resistance protein B | - | - | -3.43 |
|  | BC4959 | G | yutF | 4-nitrophenylphosphatase | - | -1.77 | **-2.66** |
|  | BC5012 | G | ydhL | Chloramphenicol resistance protein | - | **-3.39** | - |
|  | BC5030 | GER |  | Transporter, Drug/Metabolite Exporter family | - | - | **-1.83** |
|  | BC5058 | G |  | chloramphenicol resistance protein | - | - | **-1.75** |
|  | BC5114 | G |  | hypothetical protein | **-2.02** | -3.07 | **-1.71** |
|  | BC5209 | G | licH | 6-phospho-beta-glucosidase | **-1.60** | - | -1.83 |
|  | BC5211 | G |  | PTS system, lichenan oligosaccharide-specific IIC component | - | - | -3.69 |
|  | BC5258 | G |  | Phosphoglycerate transporter protein | - | -2.28 | - |
|  | BC5271 | MG |  | UDP-N-acetylglucosamine 4-epimerase | - | **-1.89** | **-4.19** |
|  | BC5274 | MG | yveM | UDP-N-acetylglucosamine 4,6-dehydratase | - | **-1.59** | **-4.39** |
|  | BC5276 | GM | ywqE | Phosphotyrosine-protein phosphatase (capsular polysaccharide biosynthesis) | - | - | **-4.84** |
|  | BC5442 | GEPR |  | Transporter, MFS superfamily | - | - | **-1.64** |
| Glycolysis (KEGG pathway) | BC1691 | G |  | Phosphoglycerate mutase | - | - | **-2.61** |
| Carbohydrate metabolism (COG category) | BC4260 | KG | glcK | Glucokinase | - | - | **-1.68** |
|  | BC4365 | C, C |  | Alcohol dehydrogenase | - | - | **-4.33** |
|  | BC4599 | G, T |  | Pyruvate kinase | - | - | **-2.26** |
|  | BC4600 | G | pfkA | 6-phosphofructokinase | - | - | **-1.81** |
|  | BC4898 | G |  | Glucose-6-phosphate isomerase | - | - | **-2.34** |
|  | BC4919 | G | yhxB | Phosphoglucomutase | - | - | **-2.01** |
|  | BC4962 | G |  | Fructose-1,6-bisphosphatase | - | - | **-2.04** |
|  | BC4996 | C | ldh | L-lactate dehydrogenase | - | -2.05 | **-2.02** |
|  | BC5135 | G | eno | Enolase | - | - | **-1.78** |
|  | BC5136 | G | pgm | Phosphoglycerate mutase | - | - | **-1.85** |
| Glycolysis Doan et al., 2003 | BC5137 | - | tpiA |  | - | - | **-1.78** |
|  | BC5138 | - | pgk |  | - | - | **-1.50** |

(-) means the gene was not significantly and highly affected in the given condition.

Genes with ratios in **bold** were restored to close to WT levels by complementation.

**Table C in S1 File. Predicted Sigma 54 regulon.** Predicted regulon members of Sigma 54 according significantly affected in the *rpoN* mutant in the transcriptomic study (p<0.01, no cutoff for expression ratio). The predicted regulon members were obtained as described in [[23](#_ENREF_23)], by in silico search of the conserved -12-24 promoter region of Sigma 54.

|  |  | | | **log2 Δ*rpoN*/WT** | | |
| --- | --- | --- | --- | --- | --- | --- |
| **Gene** | **Function** | **-12/-24 sequence** | **Distance from translation start** | **shaking t1 (mid-exp.)** | **shaking t2 (end-exp.)** | **static exp.** |
| BC3010 | collagenase | TTGGCACGGTTTTTGCT | 184 | - | - | - |
| BC4163 | phosphate butyryltransferase | TTGGCACGGTATTTGCT | 43 | **-8.54** | -5.46 | **-7.86** |
| BC2779 | acetoin dehydrogenase E1 component alpha-subunit | TTGGCACGGTACTTGCA | 36 | - | - | - |
| BC3875 | Xaa-Pro dipeptidase | CTGGCACAATTCTTGCT | 28 | - | - | **2.38** |
| BC5211 | PTS system, lichenan oligosaccharide-specific IIC component | TTGGCACGCTAATTGCA | 388 | - | - | -3.69 |
| BC2836 | sarcosine oxidase beta subunit | TTGGCACGTCAATTGCA | 40 | - | - | - |
| BC2838 | hypothetical protein | TTGGCATGATTTTTGCT | -12 | - | - | - |
| BC0153 | methionine aminopeptidase | CTGGCAGGATCGTTGCT | -63 | - | - | -1.09 |
| BC0905 | proline racemase | TTGGCATGATATTTGCA | 37 | - | - | - |
| BC2251 | lysine 2,3-aminomutase | TTGGCATAACTATTGCT | 38 | - | - | - |
| BC2194 | azoreductase | ATGGCATGACTCTTGCT | 425 | 1.13 | 1.28 | - |
| BC2335 | catabolite gene activator | CTGGCACACTAATGGCT | 143 | - | - | - |
| BC2434 | MarR family transcriptional regulator | CTGGCACGTTTTCTGCA | 386 | - | **1.79** | **1.89** |
| BC0355 | 4-aminobutyrate aminotransferase | TTGGCATATATTTTGCA | -33 | - | - | - |
| BC0107 | 2-C-methyl-D-erythritol 2,4-cyclodiphosphate synthase | TTGGCACAGATGATGCA | 240 | - | - | - |
| BC0217 | 2,5-diketo-D-gluconic acid reductase | AAGGCACGCCTGTTGCT | 451 | 1.45 | 1.43 | - |
| BC0373 | Na+/H+ antiporter NnaC | TTGGTACAACCGTTGCT | -130 | - | - | - |
| BC0474 | hypothetical protein | TTGGTACGCATTTTGCA | 78 | - | - | - |
| BC0476 | acetylornithine deacetylase | TTGGTACGCATTTTGCA | 2013 | - | - | -1.00 |
| BC0677 | undecaprenyl pyrophosphate phosphatase | TTAGCACAACATTTGCT | -114 | -0.85 | -2.01 | -2.70 |
| BC2225 | 6-phosphogluconate dehydrogenase-like protein | TTGGAACGACAATTGCA | 361 | - | - | -1.33 |
| BC2806 | acetyltransferase | TTGGCACACGTGTTTCT | 232 | - | - | - |
| BC3772 | tRNA 2-methylthioadenosine synthase | ATGGCAAGTTCATTGCT | 296 | - | - | - |
| BC3830 | tRNA (uracil-5-)-methyltransferase Gid | TTGGCGCAGGTCTTGCA | -43 | - | -0.73 | **-2.02** |
| BC5347 | putative UV damage endonuclease | TTGGAACAATATTTGCT | 45 | - | - | - |
| BC0336 | somatin-like protein | CTAGCATGAATTTTGCT | 354 | - | **-1.73** | 1.47 |
| BC0490 | hypothetical protein | TTGGAACGTATTTTGCA | 485 | - | - | - |
| BC1261 | ATP/GTP-binding protein | TTGGCACATCATTTGAT | 87 | - | - | -0.83 |
| BC1316 | PhaR protein | TTGGCATGGAGTTTGGT | 215 | 5.42 | 5.87 | 5.65 |
| BC1558 | hypothetical protein | TTGGTACATATTTTGCT | 223 | 1.47 | **2.37** | **3.20** |
| BC2740 | preprotein translocase subunit SecY | TTGGCACGTTTATTCCA | -105 | 0.95 | 0.79 | 0.23 |
| BC4424 | cysteine desulfurase | TTGGCATAATTGTTGCC | 143 | - | - | - |

(-) means the gene was not significantly affected in the given condition.

Genes with ratios in **bold** were restored to close to WT levels by complementation.

**Table D in S1 File. Aminoacid metabolism.** Genes involved in Valine, leucine and Isoleucine degradation significantly down regulated in the *rpoN* mutant

(p<0.01, ratio >3).

|  |  |  |  |  | **log2 Δ*rpoN*/WT** | | |
| --- | --- | --- | --- | --- | --- | --- | --- |
| **Biological function** | **Gene** | **COG** | **Name** | **Annotation** | **shaking t1 (mid-exp.)** | **shaking t2 (end-exp.)** | **static exp.** |
| Valine, Leucine, Isoleucine degradation (KEGG pathway) | BC4157 | C | bkdB | Lipoamide acyltransferase component of branched-chain alpha-keto acid dehydrogenase complex | **-7.05** | -4.64 | **-6.52** |
|  | BC4158 | C |  | 2-oxoisovalerate dehydrogenase beta subunit | **-7.29** | -3.93 | **-5.99** |
|  | BC4159 | C |  | 2-oxoisovalerate dehydrogenase alpha subunit | **-7.86** | -4.03 | -6.74 |
|  | BC4160 | C | lpdV | Dihydrolipoamide dehydrogenase | **-8.48** | -5.33 | -7.57 |
|  | BC4161 | C |  | Branched-chain-fatty-acid kinase | **-8.34** | -5.11 | -6.84 |
|  | BC4162 | E |  | Leucine dehydrogenase | **-5.69** | -3.09 | -5.34 |
|  | BC4163 | C |  | Phosphate butyryltransferase | **-8.54** | -5.46 | **-7.86** |

(-) means the gene was not significantly and highly affected in the given condition.

Genes with ratios in **bold** were restored to close to WT levels by complementation.

**Table E in S1 File. Motility.** Genes related to motility significantly down regulated in the *rpoN* mutant (p<0.01, ratio >3).

|  |  |  |  |  | **log2 Δ*rpoN*/WT** | | |
| --- | --- | --- | --- | --- | --- | --- | --- |
| **Biological function** | **Gene** | **COG** | **Name** | **Annotation** | **shaking t1 (mid-exp.)** | **shaking t2 (end-exp.)** | **static exp.** |
| Motility genes (COG category) | BC0404 | NT |  | Methyl-accepting chemotaxis protein | - | - | **-2.13** |
|  | BC0422 | NT |  | Methyl-accepting chemotaxis protein | - | -3.88 | **-5.48** |
|  | BC0559 | NT |  | Methyl-accepting chemotaxis protein | - | -3.12 | **-3.74** |
|  | BC0576 | NT |  | Methyl-accepting chemotaxis protein | -1.83 | -2.90 | **-3.00** |
|  | BC0678 | NT |  | Methyl-accepting chemotaxis protein | - | **-2.98** | **-3.57** |
|  | BC1124 | NT |  | Methyl-accepting chemotaxis protein | - | - | **-2.66** |
|  | BC1625 | N |  | Chemotaxis motA protein | - | **-3.01** | **-5.09** |
|  | BC1626 | N | ytxE | Chemotaxis motB protein | - | **-1.71** | **-2.49** |
|  | BC1628 | NT | cheA | Chemotaxis protein cheA | - | **-2.26** | **-4.94** |
|  | BC1629 | NT, NU | fliY | Chemotaxis protein cheC | - | - | **-3.02** |
|  | BC1632 | NT | cheR | Chemotaxis protein methyltransferase | - | - | **-1.72** |
|  | BC1636 | N |  | Flagellar hook-associated protein 1 | - | **-1.80** | **-3.54** |
|  | BC1637 | N |  | Flagellar hook-associated protein 3 | - | **-1.61** | **-3.09** |
|  | BC1638 | N |  | Flagellar hook-associated protein 2 | - | - | **-3.27** |
|  | BC1639 | NUO |  | Flagellar protein fliS | - | - | **-2.85** |
|  | BC1641 | N |  | Flagellar basal-body rod protein flgB | - | **-1.87** | **-3.72** |
|  | BC1642 | N | flgC | Flagellar basal-body rod protein flgC | - | **-1.89** | **-3.72** |
|  | BC1643 | NU |  | Flagellar hook-basal body complex protein fliE | - | **-2.10** | **-4.10** |
|  | BC1644 | NU |  | Flagellar M-ring protein fliF | - | - | **-3.59** |
|  | BC1645 | N | fliG | Flagellar motor switch protein fliG | - | - | **-3.46** |
|  | BC1647 | NU |  | Flagellum-specific ATP synthase | - | - | **-4.05** |
|  | BC1650 | N |  | Basal-body rod modification protein flgD | - | - | **-3.05** |
|  | BC1651 | N |  | Flagellar hook protein flgE | - | - | **-3.46** |
|  | BC1654 | T, NT | cheV | Chemotaxis protein cheV | - | - | **-3.66** |
|  | BC1656 | N |  | Flagellin | - | - | **-4.97** |
|  | BC1658 | N | yvzB | Flagellin | - | -2.19 | **-4.91** |
|  | BC1661 | NU |  | Flagellar motor switch protein fliN | - | - | **-2.64** |
|  | BC1662 | N | fliM | Flagellar motor switch protein fliM | - | - | **-2.89** |
|  | BC1663 | NU |  | Flagellar motor switch protein fliN | - | - | **-2.45** |
|  | BC1665 | NU | fliP | Flagellar biosynthetic protein fliP | - | - | **-3.25** |
|  | BC1666 | NU |  | Flagellar biosynthetic protein fliQ | - | - | **-3.06** |
|  | BC1667 | NU | fliR | Flagellar biosynthetic protein fliR | - | - | **-2.97** |
|  | BC1668 | NU | flhB | Flagellar biosynthetic protein flhB | - | - | **-2.71** |
|  | BC1671 | N | flhA | Flagellar basal-body rod protein flgG | - | **-1.88** | **-3.77** |
|  | BC2006 | NT | tlpA | Methyl-accepting chemotaxis protein | - | - | **-3.41** |
|  | BC3101 | NT |  | Hemolysin BL binding component precursor | -1.89 | -3.15 | **-1.60** |
|  | BC3520 | NT |  | Methyl-accepting chemotaxis protein | - | - | **-2.82** |
|  | BC4512 | N | motB | Chemotaxis motB protein | - | **-2.96** | **-4.44** |
|  | BC4513 | N | motA | Chemotaxis motA protein | - | **-2.12** | **-2.80** |
|  | BC5034 | NT | yoaH | Methyl-accepting chemotaxis protein | - | -3.50 | **-4.26** |
|  | BC5065 | NT |  | Methyl-accepting chemotaxis protein | - | - | **-1.87** |
|  | BC5424 | NT |  | Methyl-accepting chemotaxis protein | - | - | **-1.85** |

(-) means the gene was not significantly and highly affected in the given condition.

Genes with ratios in **bold** were restored to close to WT levels by complementation.

**Table F in S1 File. Biofilm formation.** Genes related to biofilm formation significantly affected in the *rpoN* mutant (p<0.01, ratio >3).

|  |  |  |  |  | **log2 Δ*rpoN*/WT** | | |
| --- | --- | --- | --- | --- | --- | --- | --- |
| **Biological function** | **Gene** | **COG** | **Name** | **Annotation** | **shaking t1 (mid-exp.)** | **shaking t2 (end-exp.)** | **static exp.** |
| CPS cluster genes (Ivanova et al., 2003) | BC5263 | M |  | UDP-glucose 4-epimerase | - | **-2.53** | **-3.60** |
|  | BC5264 |  |  | EPSX protein | - | **-2.52** | **-3.65** |
|  | BC5265 | K | lytR | Transcriptional regulator, LytR family | - | **-1.84** | **-1.75** |
|  | BC5266 | R |  | Heteropolysaccharide repeat unit export protein | - | **-2.23** | **-3.05** |
|  | BC5267 | M | yveT | Glycosyltransferase | - | **-1.68** | **-2.52** |
|  | BC5268 |  |  | Secreted polysaccharide polymerase | - | **-1.84** | **-2.98** |
|  | BC5269 | M |  | Amylovoran biosynthesis AmsK | - | **-2.42** | **-3.97** |
|  | BC5270 | M | yvfC | Undecaprenyl-phosphate galactosephosphotransferase | - | **-2.21** | **-4.32** |
|  | BC5271 | MG |  | UDP-N-acetylglucosamine 4-epimerase | - | **-1.89** | **-4.19** |
|  | BC5272 |  |  | Carbamoyl-phosphate synthase small chain | - | - | **-3.86** |
|  | BC5273 | M | spsC | UDP-bacillosamine synthetase | - | **-1.82** | **-4.89** |
|  | BC5274 | MG | yveM | UDP-N-acetylglucosamine 4,6-dehydratase | - | **-1.59** | **-4.39** |
|  | BC5275 | M |  | UTP--glucose-1-phosphate uridylyltransferase | - | - | **-2.65** |
|  | BC5276 | GM | ywqE | Phosphotyrosine-protein phosphatase (capsular polysaccharide biosynthesis) | - | - | **-4.84** |
|  | BC5277 | D | ywqD | Tyrosine-protein kinase (capsular polysaccharide biosynthesis) | - | **-2.47** | **-5.53** |
|  | BC5278 | M | ywqC | Chain length regulator (capsular polysaccharide biosynthesis) | - | **-2.40** | **-5.71** |
|  | BC5279 | D |  | Tyrosine-protein kinase (capsular polysaccharide biosynthesis) | - | **3.44** | **3.07** |
| Biofilm regulator (Kearns et al., 2005) | BC1282 | K | sinR | SinR protein | 1.63 | - | **2.59** |
|  | BC1283 |  | sinI | SinI protein | 1.83 | 1.95 | 5.04 |
| EPS production (Gao et al., 2015) | BC1278 | U | sipW | Signal peptidase I | - | - | **-1.6** |

(-) means the gene was not significantly and highly affected in the given condition.

Genes with ratios in **bold** were restored to close to WT levels by complementation.

**Table G in S1 File. Virulence.** Genes related to virulence significantly affected in the *rpoN* mutant (p<0.01, ratio >3).

|  |  |  |  |  | **log2 Δ*rpoN*/WT** | | |
| --- | --- | --- | --- | --- | --- | --- | --- |
| **Biological function** | **Gene** | **COG** | **Name** | **Annotation** | **shaking t1 (mid-exp.)** | **shaking t2 (end-exp.)** | **static exp.** |
| Virulence (PlcR regulon, Gohar et al., 2008) | BC1809 | - | nheA | Non-hemolytic enterotoxin lytic component L2 | **-2.33** | -4.53 | **-1.97** |
|  | BC1810 | - | nheB | Non-hemolytic enterotoxin lytic component L1 | -2.71 | -4.46 | **-1.79** |
|  | BC1811 | D | nheC | Non-expressed Enterotoxin C | -2.35 | **-2.56** | - |
|  | BC3102 | - | hblB | Hemolysin BL binding component precursor | -1.62 | -2.25 | -1.96 |
|  | BC3103 | - | hblL1 | Hemolysin BL lytic component L1 | -1.90 | -2.57 | -2.18 |
|  | BC3104 | - | hblL2 | Hemolysin BL lytic component L2 | -1.74 | -2.14 | **-1.79** |
|  | BC5101 | - | clo | Perfringolysin O precursor | -2.31 | -4.00 | - |
|  | BC1110 | - | cytK | Cytotoxin K | - | -5.30 | **-2.33** |
|  | BC3761 | - | plcA | 1-phosphatidylinositol phosphodiesterase precursor | - | -3.59 | - |
|  | BC0670 | - | plcB | Phospholipase C | -2.30 | -4.87 | **-2.49** |
|  | BC0671 | - | smase | Sphingomyelin phosphodiesterase | - | -4.38 | **-1.72** |
|  | BC2735 | E | nprP2 | Bacillolysin | - | - | - |
|  | BC3383 | E | nprC | Bacillolysin | - | **-1.74** | **-1.70** |
|  | BC5351 | E | nprB | Bacillolysin | - | -3.68 | **1.84** |
|  | BC0556 | R | colC | Microbial collagenase | - | -1.65 | - |
|  | BC3161 | R | colA | Microbial collagenase | **-2.37** | - | - |
|  | BC3384 | - | mpbE | Enhancin | - | - | - |
|  | BC3762 | O | sfp | Microbial collagenase | **-2.02** | -4.10 | **-3.45** |
|  | BC5101 | - | sppc1 | Perfringolysin O precursor | -2.31 | -4.00 | - |
|  | BC2463 |  | sppc2 | Peptide with anti-bacterial activity | - | - | - |
|  | BC3185 | - | sppc3 | hypothetical protein | - | - | - |
|  | BC5349 | - | papR | PapR protein | - | **-1.81** | - |
|  | BC0576 | NT | mcpA | Methyl-accepting chemotaxis protein | -1.83 | -2.90 | **-3.00** |
|  | BC3385 | NT | tlpA | Methyl-accepting chemotaxis protein | - | - | - |
|  | BC0577 | T | yufL | Two-component sensor kinase yufL | - | - | - |
|  | BC3747 | T, T |  | Sensory box/GGDEF family protein | - | -2.27 | - |
|  | BC4509 | CP | yhaP | Sodium export permease protein | - | -2.07 | **-2.03** |
|  | BC4510 | R | yhaQ | Sodium export ATP-binding protein | - | **-2.13** | **-1.86** |
|  | BC2411 | GEPR |  | Macrolide-efflux protein | - | - | - |
|  | BC3763 | - | cwh | Cell wall hydrolase | - | **-2.01** | **-3.08** |
|  | BC0991 | D | slpA | S-layer homology domain / putative murein endopeptidase | **-2.26** | -5.53 | **-5.50** |
|  | BC3746 | R | Predicted | 2-hydroxy-6-oxo-6-phenylhexa-2,4-dienoate hydrolase | - | - | - |
|  | BC0666 | S | inhA2 | Immune inhibitor A precursor | - | - | - |
|  | BC4999 | R |  | CAAX amino terminal protease family | - | -2.54 | **-2.25** |
|  | BC4511 | R | lppC | Acid phosphatase | **-1.89** | -5.31 | **-3.65** |
|  | BC2552 | - |  | hypothetical protein | - | **-2.09** | **-3.39** |
|  | BC1713 | - |  | hypothetical Membrane Spanning Protein | **-1.71** | **-2.79** | - |
|  | BC3527 | - |  | hypothetical protein | - | **-2.06** | **1.77** |
|  | BC0361 | G | yxkH | Polysaccharide deacetylase | - | - | - |
|  | BC0362 | - |  | hypothetical protein | - | - | - |
|  | BC0578 | KT | yufM | Two-component response regulator yufM | - | - | - |
|  | BC2410 | K | tetR | Transcriptional regulator, TetR family | - | - | - |
|  | BC1082 | KR |  | Ribosomal-protein-alanine acetyltransferase | - | - | - |
|  | BC5350 | R, K | plcR | Transcriptional activator plcR | - | **-2.94** | **-2.22** |
|  | BC1081 | - | prp2 | PlcR-regulated protein PRP2 | - | **-3.67** | **-2.51** |

(-) means the gene was not significantly and highly affected in the given condition.

Genes with ratios in **bold** were restored to close to WT levels by complementation.

**Table H in S1 File. Anaerobic respiration.** Genes relevant for anaerobic respiration significantly down regulated in the *rpoN* mutant (p<0.01, ratio >3)..

|  |  |  |  |  | **log2 Δ*rpoN*/WT** | | |
| --- | --- | --- | --- | --- | --- | --- | --- |
| **Biological function** | **Gene** | **COG** | **Name** | **Annotation** | **shaking t1 (mid-exp.)** | **shaking t2 (end-exp.)** | **static exp.** |
| Anaerobic respiration (Nakano et al, 1996) | BC1477 | TK | resD | Transcriptional regulatory protein resD | - | - | **-2.30** |
|  | BC1478 | T | resE | Sensor protein resE | - | - | **-2.28** |
|  | BC2118 | C | narG | Respiratory nitrate reductase alpha chain | - | - | -3.74 |
|  | BC2119 | C | narH | Respiratory nitrate reductase beta chain | - | - | -5.98 |
|  | BC2120 | C | narJ | Respiratory nitrate reductase delta chain | - | - | -5.33 |
|  | BC2121 | C | narI | Respiratory nitrate reductase gamma chain | - | - | -5.54 |
|  | BC2122 | T | fnr | Transcription regulator, Crp family | - | - | - |
|  | BC2128 | P | narK | Nitrite extrusion protein | - | - | -7.64 |
|  | BC2135 | PR | nasE | Nitrite reductase [NAD(P)H] small subunit | - | - | -6.23 |
|  | BC2136 | C | nasD | Nitrite reductase [NAD(P)H] large subunit | **-2.37** | - | -5.52 |

(-) means the gene was not significantly and highly affected in the given condition.

Genes with ratios in **bold** were restored to close to WT levels by complementation.

**Table I in S1 File. Regulators** **and sporulation related genes.** Sigma factors significantly affected in the *rpoN* mutant (p<0.01, ratio >3).

|  |  |  |  |  | **Log2 Δ*rpoN*/WT** | | |
| --- | --- | --- | --- | --- | --- | --- | --- |
| **Biological function** | **Gene** | **COG** | **Name** | **Annotation** | **shaking t1 (mid-exp.)** | **shaking t2 (end-exp.)** | **static exp.** |
| Sigma factors | BC0647 | K |  | RNA polymerase ECF-type sigma factor | **3.51** | **3.30** | - |
|  | BC1002 | T | rsbV | Anti-sigma B factor antagonist | - | - | **2.38** |
|  | BC1003 | T | rsbW | Anti-sigma B factor | - | - | **3.00** |
|  | BC1004 | K | sigB | RNA polymerase sigma-B factor | - | - | **1.94** |
|  | BC1114 | K | sigM | RNA polymerase sigma factor sigM | - | - | **1.97** |
|  | BC2108 | K | sigZ | RNA polymerase ECF-type sigma factor | - | - | **2.51** |
|  | BC2386 | K |  | RNA polymerase ECF-type sigma factor | 3.97 | **2.67** | **2.10** |
|  | BC3426 | K | sigI | RNA polymerase sigma-I factor | 4.81 | **1.98** | **2.07** |
|  | BC5143 | K | sigL | RNA polymerase sigma-54 factor rpoN | -6.04* | -3.7* | -4.85* |
|  | BC5363 | K | sigW | RNA polymerase ECF-type sigma factor | - | - | **3.73** |
| Sporulation related | BC0042 | K | abrB | Transcription state regulatory protein abrB | - | **-2.4** | **-** |
|  | BC2142 | S |  | Stage V sporulation protein S | **3.5** | **3.6** | 4.3 |

* Expression of the rpoN gene was restored in the complemented mutant, but in shaking t1 (mid-exp.) and static exp. it was higher than in the WT, and in shaking t2 (end-exp.) it was closer to WT but still significantly different from it.

(-) means the gene was not significantly and highly affected in the given condition.

Genes with ratios in **bold** were restored to close to WT levels by complementation.
